# Analysis of Two Putative C*andida albicans* Phosphopantothenoylcysteine Decarboxylase / Protein Phosphatase Z Regulatory Subunits Reveals an Unexpected Distribution of Functional Roles

**DOI:** 10.1371/journal.pone.0160965

**Published:** 2016-08-09

**Authors:** Katalin Petrényi, Cristina Molero, Zoltán Kónya, Ferenc Erdődi, Joaquin Ariño, Viktor Dombrádi

**Affiliations:** 1 Department of Medical Chemistry, Faculty of Medicine, University of Debrecen, 4032 Debrecen, Hungary; 2 Institut de Biotecnologia i Biomedicina and Departament de Bioquímica i Biologia Molecular, Universitat Autònoma de Barcelona, Cerdanyola del Vallès 08193, Barcelona, Spain; Louisiana State University, UNITED STATES

## Abstract

Protein phosphatase Z (Ppz) is a fungus specific enzyme that regulates cell wall integrity, cation homeostasis and oxidative stress response. Work on *Saccharomyces cerevisiae* has shown that the enzyme is inhibited by Hal3/Vhs3 moonlighting proteins that together with Cab3 constitute the essential phosphopantothenoylcysteine decarboxylase (PPCDC) enzyme. In *Candida albicans* CaPpz1 is also involved in the morphological changes and infectiveness of this opportunistic human pathogen. To reveal the CaPpz1 regulatory context we searched the *C*. *albicans* database and identified two genes that, based on the structure of their *S*. *cerevisiae* counterparts, were termed CaHal3 and CaCab3. By pull down analysis and phosphatase assays we demonstrated that both of the bacterially expressed recombinant proteins were able to bind and inhibit CaPpz1 as well as its C-terminal catalytic domain (CaPpz1-Cter) with comparable efficiency. The binding and inhibition were always more pronounced with CaPpz1-Cter, indicating a protective effect against inhibition by the N-terminal domain in the full length protein. The functions of the *C*. *albicans* proteins were tested by their overexpression in *S*. *cerevisiae*. Contrary to expectations we found that only CaCab3 and not CaHal3 rescued the phenotypic traits that are related to phosphatase inhibition by ScHal3, such as tolerance to LiCl or hygromycin B, requirement for external K^+^ concentrations, or growth in a MAP kinase deficient *slt2* background. On the other hand, both of the *Candida* proteins turned out to be essential PPCDC components and behaved as their *S*. *cerevisiae* counterparts: expression of CaCab3 and CaHal3 rescued the *cab3* and *hal3 vhs3 S*. *cerevisiae* mutations, respectively. Thus, both CaHal3 and CaCab3 retained the PPCDC related functions and have the potential for CaPpz1 inhibition *in vitro*. The fact that only CaCab3 exhibits its phosphatase regulatory potential *in vivo* suggests that in *C*. *albicans* CaCab3, but not CaHal3, acts as a moonlighting protein.

## Introduction

The *Saccharomyces cerevisiae SIS2*/*HAL3* gene was discovered as a suppressor of the *sit4* mutation [1] and a regulator of salt tolerance [2]. These apparently unrelated functions were clarified when it was demonstrated that Hal3 acts as a negative regulatory subunit of the Ppz1 Ser/Thr protein phosphatase [3–5]. Ppz1 has a primary role in regulating monovalent cation homeostasis in two different ways: inhibiting the influx of potassium (through the Trk high-affinity potassium transporters) and inhibiting the efflux of sodium (by downregulating the expression of the *ENA1* ATPase) [5–7]. Subsequent work identified the gene *VHS3* (a *HAL3* paralog that arose from the whole genome duplication) encoding a second inhibitory subunit of Ppz1 [8]. Both ScHal3 and ScVhs3 bind to the catalytic, C-terminal domain of Ppz1 [3,8] with a 1:1 stoichiometry [9].

**Fig 1:**
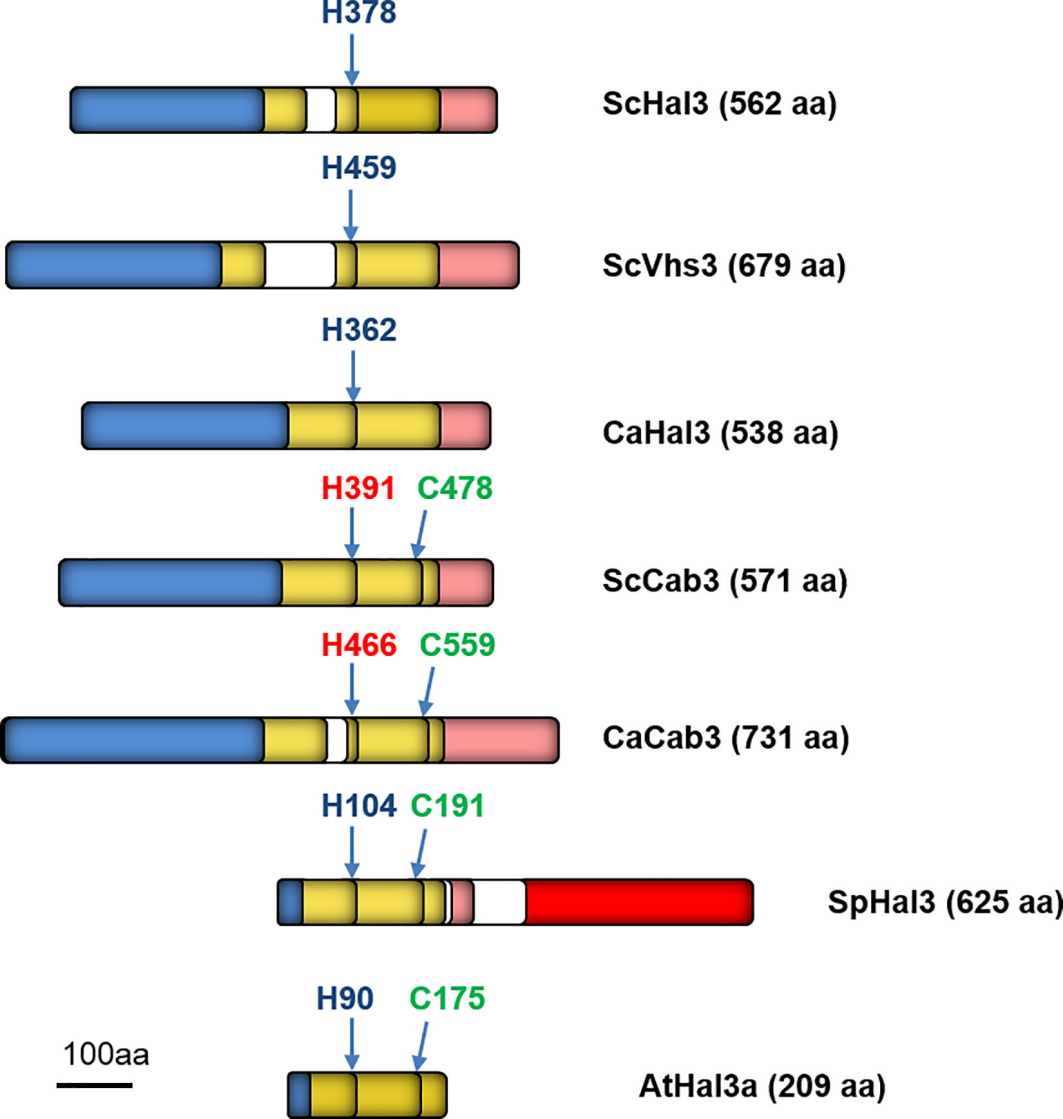
Schematic representation of the primary structure of typical Hal3 orthologs in fungi. The amino acid sequences of *S*. *cerevisiae* ScHal3, ScVhs3, and ScCab3; *C*. *albicans* CaHal3 and CaCab3; *S*. *pombe* SpHal3; and *A*. *thaliana* AtHal3a were aligned by the Clustal W software along the conserved positions of the essential His (blue) and Cys (green) residues that are indispensable for PPCDC activity. The conserved, but nonessential His residues are depicted in red. The N-terminal intrinsically disordered segments of the proteins are blue, the PD domain required for the decarboxylase activity is yellow, and the disordered acidic segments (mainly at the C-terminus) are pink. The nonhomologous regions are white, while the red bar indicates the fusion of a C-terminal thymidylate synthase domain in SpHal3. The scale bar indicates the size of a 100 amino acid long protein segment. The number of amino acid residues in each protein is given in parenthesis.

Remarkably, while Ppz enzymes are found only in fungal species, orthologs of *HAL3* were identified both in prokaryotic and eukaryotic organisms. This ubiquitous distribution was explained by the fact that ScHal3 and ScVhs3 are moonlighting proteins that, in *S*. *cerevisiae*, constitute two subunits of a heterotrimeric phosphopantothenoylcysteine decarboxylase (PPCDC) enzyme [10]. PPCDC catalyzes a key decarboxylation step in CoA biosynthesis. Such heterotrimer would be composed from a constant ScCab3 (a ScHal3 and ScVhs3 paralog) subunit and two ScHal3, two ScVhs3 or one of each (Fig 1). This structure explains the essential nature of *CAB3* and the synthetically lethal phenotype of the *hal3 vhs3* mutations [8,10]. It is important to note that a single ScHal3, ScVhs3 or ScCab3 polypeptide chain would not be sufficient to provide the decarboxylase activity since the active site is located at the interface of the subunits of the oligomeric enzyme. Based on the 3D structures of *A*. *thaliana* AtHal3a [11,12] and *Homo sapiens* HsCoac [13] orthologs it was proposed that in the active *S*. *cerevisiae* PPCDC holoenzyme the essential Cys478 residue of the catalytic cleft and the conserved Asn442 residue, which is involved in binding the carboxylate group of PPC, is provided by ScCab3 (Fig 1). The other half of the catalytic site, containing an essential His378 residue, must be supplied by ScHal3 or ScVhs3 (His466), since the equivalent His residue in ScCab3 (His391) is not functional [10]. ScCab3 has some affinity for ScPpz1, but cannot inhibit its phosphatase activity either *in vitro* or *in vivo* [10].

It must be noted that the *S*. *cerevisiae* PPCDC subunit composition is exceptional, since in most of the eukaryotic organisms like *A*. *thaliana* the PPCDC activity is associated with a homotrimer lacking the N-terminal extension and the acidic C-terminal tail that is found in the investigated fungal orthologs (Fig 1). In ScHal3, this central domain, named ScHal3 PD, is required for Ppz1 binding and regulation, although the acidic C-terminal tail also plays an important role [14,15]. The examination of fungal genomes indicates that these organisms offer an intriguing variety of possibilities concerning Hal3, Vhs3 or Cab3 structures. For instance, in the fission yeast *Schizosaccharomyces pombe* the only gene product SpHal3 is composed of an N-terminal PPCDC core (with a short acidic tail) followed by a C-terminal thymidylate synthase domain (Fig 1), which arose likely in a gene fusion event. The *S*. *pombe* gene is expressed as a single polypeptide whose isolated N-terminal domain is able to inhibit ScPzh1 and SpPPz1 and provides full PPCDC activity, whereas the C-terminal half exhibits thymidylate synthase function [16].

The filamentous fungus *C*. *albicans* represents yet another paradigm for the evolution of PPCDC subunits. The analysis of the *C*. *albicans* database http://www.candidagenome.org/ revealed two genes that most likely encode the orthologs of ScHal3. The conceptual protein product of orf19.7378 is more similar to ScHal3 than to ScCab3 (36.1% vs. 25.7% identity) and contains the typical active His in the conserved environment. On the other hand the putative gene product of orf19.3260 is more reminiscent to ScCab3 (31.8% vs. 25.4% identity) and contains the conserved Cys. Based on these structural similarities, and on the phylogenetic relationships of the two predicted *Candida* proteins to the known PPCDC subunits (S1 Fig) we termed the two gene products CaHal3 and CaCab3, respectively (Fig 1). Our initial working hypothesis was that CaHal3 is one of the essential PPCDC subunits and at the same time acts as an inhibitor of CaPpz1 enzyme, while CaCab3 is the other essential PPCDC subunit that does not affect phosphatase activity. Following these lines we have investigated the properties and functions of the two putative *C*. *albicans* PPCDC subunits by biochemical and molecular genetics methods, placing a major emphasis on the fungus specific moonlighting activities, i.e. on the possible regulation of CaPpz1 functions. Our results shed light on an unexpected scenario in which CaCab3, and not CaHal3, is likely to display the moonlighting function in this opportunistic pathogenic fungal species.

## Materials and Methods

### Materials

The enzymes used in the present study were obtained from the following suppliers: restriction endonucleases (Thermo Scientific or Promega), Phire and Phusion DNA polymerases (Thermo Scientific), DNA ligase (Promega). PageRuler Prestained Protein Ladder (Thermo Scientific) and 1kb DNA standard (Invitrogen) molecular size markers were used in gel electrophoretic separations. IPTG was from Sigma Aldrich, while the other reagents were purchased from VWR Hungary.

### Fungal strains and growth conditions

All of the fungal strains used in our study are described in Table 1. Strain CCV186 was constructed by transformation of strain JC010 (*slt2*::*LEU2*) with a 2.0 kbp *hal3*::*kanMX4* cassette amplified from the *hal3* deletant in the BY4741 background with oligonucleotides 5' Hal3-200 and Hal3-3'_term1 (S1 Table). *S*. *cerevisiae* cells were grown at 28°C in YPD medium (10g l^-1^ yeast extract, 20g l^-1^ peptone and 20 g l^-1^ dextrose) or, when containing plasmids, in synthetic minimal medium lacking the appropriate selection agents [17]. *C*. *albicans* species were grown in YPD at 37°C as described before [18].

**Table 1:** Fungal strains used in the present study.

Species	Strain	Genotype	Reference
*C*. *albicans*	SN87	*ura3Δ-iro1Δ*::*imm*^*434*^*/URA3-IRO1*, *his1Δ /his1Δ*, *leu2Δ /leu2Δ*	[19]
*S*. *cerevisiae*	BY4741	*MATa his3 Δ 1 leu2 Δ met15Δ ura3Δ*	[20]
*S*. *cerevisiae*	*ppz1*::*KanMX4*	BY4741 *ppz1*::*kanMX4*	[20]
*S*. *cerevisiae*	*hal3*::*KanMX4*	*BY4741 hal3*::*KanMX4*	[20]
*S*. *cerevisiae*	MAR25	*MAT****a****/α ura3-52 leu2-3*,*112 his4 trp1-1 can-1r*, *cab3*::*KanMX4/CAB3*	[10]
*S*. *cerevisiae*	AGS4	*MATa/a ura3-52 leu2-3*,*112 his4 trp1-1 can-1r*, *hal3*::*LEU2/HAL3 vhs3*::*KanMX4/VHS3*	[8]
*S*. *cerevisiae*	JA-100	*MAT* ***a*** *leu2-3*,*112 ura3-52 trp1-1 his4 can1r (ssd1v)*	[3]
*S*. *cerevisiae*	JC010	JA-100 *slt2*::*LEU2*	[21]
*S*. *cerevisiae*	CCV186	JA-100 *slt2*::*LEU2 hal3*::*KANMX4*	this work

### DNA cloning

The pGEX-6P-1 (Amersham Biosciences) plasmid was used to express N-terminal GST tagged recombinant proteins in *E*. *coli*. Genomic DNA was isolated from the SN87 *C*. *albicans* strain by the method of Lee et al. [22]. Intronless ORFs of *CaHAL3* (orf19.7378) or *CaCAB3* (orf19.3260) were amplified from genomic DNA with CaHal3EcoRI-CaHal3XhoI or CaCAB3EcoRI-CaCAB3XhoI primer pairs (S1 Table), respectively. PCR products were digested with EcoRI and XhoI and were cloned into the pGEX-6P-1 plasmid *via* the appropriate restriction sites. CaPpz1 and its catalytic domain (CaPPZ1-Cter) coding regions were amplified with CaPpz1EcoRI-CaPpz1XhoI and CaPPZ1CterBamHI-CaPPZ1CterXhoI primer pairs (S1 Table), respectively, from the pET28-CaPPZ1 template that was described previously [18]. The oligonucleotide primers used for the cloning experiments are summarized in S1 Table. The pGEX-ScPPZ1 and pGEX-ScPPZ1-Cter plasmids coding for the full ScPpz1 protein or for its C-terminal catalytic domain (ScPpz1-Cter) were prepared according to Ruiz et al. [8]. We utilized the pWS93 high-copy number plasmid [23] for the expression of recombinant proteins with HA (haemagglutinin)-tag in *S*. *cerevisiae* under the control of the strong *ADH1* promoter. To this end, *CaHAL3* and *CaCAB3* ORFs were transferred from the pGEX6p-1-CaHAL3 and pGEX6p-1-CaCAB3 vectors into the EcoRI/SalI sites of pWS93. The generation of pWS93-ScHAL3 and pWS93-ScCAB3 plasmid was reported earlier [10].

The gel extraction kit of Qiagen and the EZ-10 spin column plasmid DNA miniprep kit of Bio Basic were applied in DNA purifications. All of the novel constructs were confirmed with DNA sequencing by UD-GenoMed Medical Genomic Technologies Ltd. (Hungary).

### Expression and purification of recombinant proteins

BL21 DE3 RIL *E*. *coli* (Stratagene) cells were used to express GST-ScPpz1, GST-ScPpz1-Cter and 6xHis-CaPpz1 as previously reported [9,18]. The protein phosphatases GST-CaPpz1 and GST-CaPpz1-Cter were expressed in the presence of 0.5 mM MnCl_2_ and were purified in the presence of 1 mM MnCl_2_ in order to preserve enzyme activity. Optimal conditions for the production of soluble proteins were determined as follows: GST-CaHal3 was expressed in the presence of 0.6 mM IPTG at 25°C for 3h; GST-CaCab3 was generated with 0.5 mM IPTG at 37°C for 3h; 0.6 mM IPTG at 18°C for 3h was used to express GST-CaPpz1; and GST-CaPpz1-Cter was produced under identical conditions overnight. The N-terminal GST-tagged proteins were purified by affinity chromatography on gluthation-Sepharose 4B resin (GE Healthcare) according to the manufacturer´s instructions, and, when required, their GST-tag was removed with PreScission protease (GE Healthcare), as described [24]. Protein concentration was measured [25] with BSA as standard, and SDS-PAGE was performed as previously described [26]. The SDS-PAGE analysis of all recombinant proteins used in the present study is presented in S2 Fig. Gels were scanned with Alpha Innotech Fluorchem FC2 apparatus and protein purity was estimated with ImageJ software.

### Detection of protein-protein interactions

The *in vitro* interaction between purified recombinant proteins was tested by pull down method in two ways. In the first experiment 1 μg of gluthation-Sepharose 4B bound GST-CaPpz1-Cter was incubated for 30–40 min at 24°C with 4 μg of purified CaHal3, CaCab3 or ScHal3 proteins. The unbound proteins were washed off and the matrix bound proteins were analyzed by SDS-PAGE. Alternatively GST-CaHal3, GST-CaCab3 or GST (negative control) were immobilized to gluthation-Sepharose 4B, and then incubated with 6xHis-CaPpz1 protein phosphatase for 30 min at room temperature. The resins were washed four times with 200 μl elution buffer (50 mM Tris-HCl pH 7.5, 150 mM NaCl). The bound proteins were removed from the matrix with 1xSDS-PAGE loading buffer by boiling for 5 min. Proteins were separated by SDS-PAGE, and were transferred onto nitrocellulose Hybond ECL membrane (GE Healthcare). After blocking with 5% milk powder in TBST (20 mM Tris, pH 7.5, 500 mM NaCl, 0.05% Tween20 and 0.2% Triton X-100) the membrane was incubated with the anti-6xHis primary antibody (Qiagen, 1:1000 dilution) overnight at 4°C. After washing, a peroxidase conjugated anti mouse secondary antibody (Sigma, 1:3000 dilution) was added and incubated for one hour. Immunoreactive proteins were visualized with ECL reagents (Thermo Scientific) on X-ray film (Kodak).

In the second set of experiments HA-tagged recombinant interacting proteins were expressed in the *S*. *cerevisiae ppz1Δ* strain. The mutant yeast cells were transformed either with empty pWS93 (negative control) or with pWS93-ScHal3, pWS93-CaHal3 and pWS93-CaCab3 vectors. Yeast extracts were prepared as previously described [15]. 150 μg of each protein extract were mixed with 50 μl of gluthation-Sepharose 4B resin containing ether immobilized GST-CaPpz1 or immobilized GST. The interactions were analyzed as in [15] with the exception that we used anti-HA primary antibody from Invitrogen (1:1000 dilution) overnight, that was followed by the incubation with peroxidase conjugated rabbit secondary antibody (Sigma,1:5000 dilution) for one hour.

### Assay of protein phosphatase activity

The sensitive radioactive assay described in [27] was adopted to determine the activity of recombinant CaPpz1, CaPpz1-Cter, ScPpz1 and ScPpz1-Cter phosphatases. The enzyme activity was measured with 1 μM [^32^P]-labeled 20 kDa turkey gizzard myosin light (^32^P-MLC20) chain substrate in the presence of 2 mM MnCl_2_.

### Phenotypic analysis of yeast cells

Sensitivity of *S*. *cerevisiae* cells to LiCl (Reanal), caffeine (Merck), and hygromycin (Sigma) was evaluated by growth on agar plates (drop test) as described previously [5]. Given the unexpected results of these tests, we confirmed the nature of the plasmid by colony PCR. Single colonies were picked after the completion of the growth tests, and were analyzed by PCR using either the CaHal3EcoRI-CaHal3XhoI or the CaCab3EcoRI-CaCab3XhoI primer pairs (S1 Table). Growth under limiting external potassium concentrations was performed using Translucent K^+^-free medium [28], which contains a negligible amount (15 μM) of potassium ions, supplemented with 1 mM or 50 mM KCl, as previously reported [29].

### Tetrad analysis

For sporulation of *S*. *cerevisiae* diploid strains, cells were transferred to liquid medium containing 10 g l^-1^ potassium acetate, 1g l^-1^ yeast extract and 0.5 g l^-1^ glucose (pH 7.2) for several days. Tetrad analysis was carried out as previously described [30]. Asci were dissected using the MSM 300 Yeast Dissection Microscope (Singer Instruments). To determine the genotype of each spore, haploid colonies were replicated into plates with media selecting for the appropriate genetic markers.

## Results and Discussion

### Interaction of the CaHal3 and CaCab3 proteins with the CaPpz1 phosphatase

**Fig 2:**
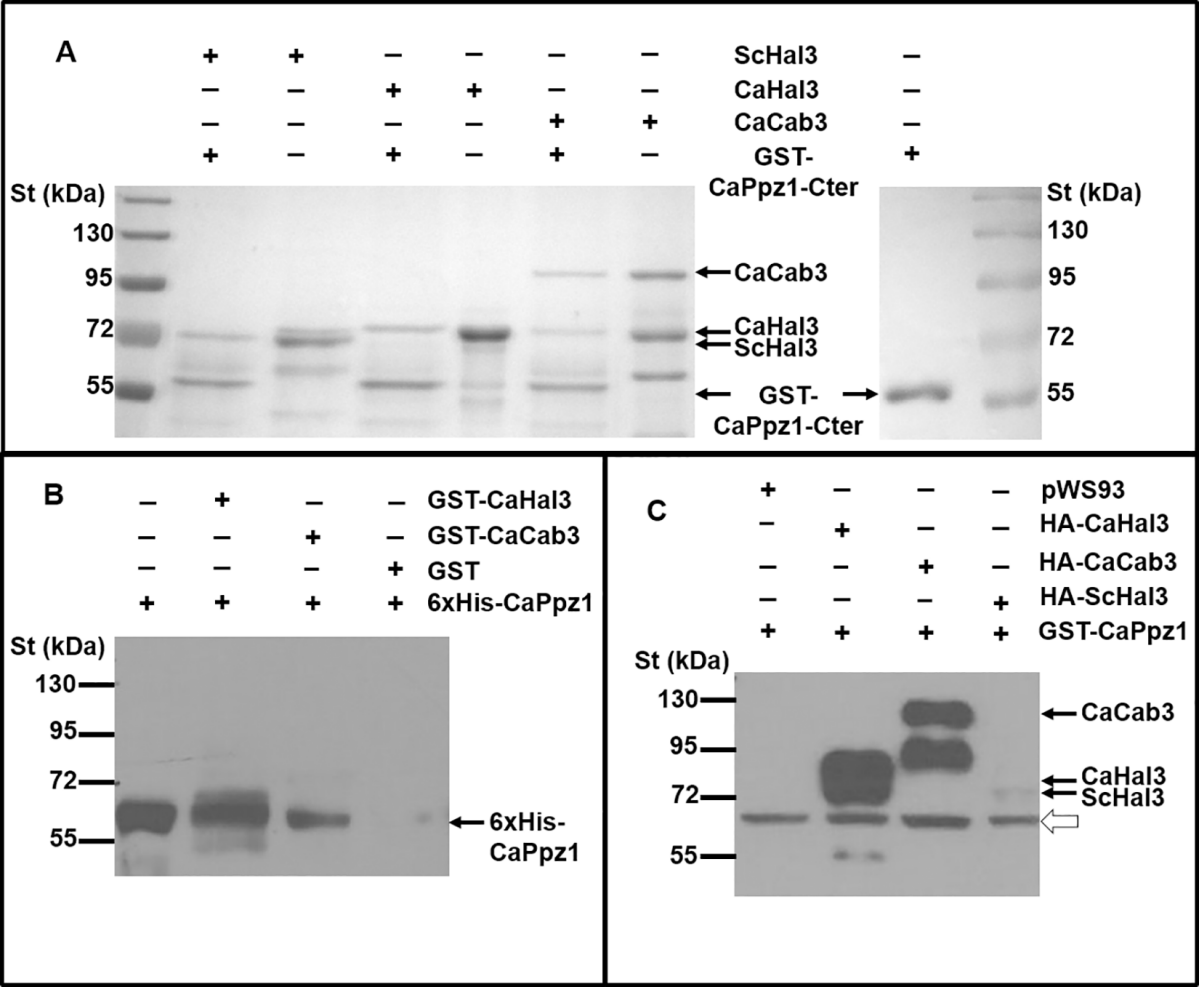
Interaction between CaPpz1 or its catalytic domain, CaPpz1-Cter, with Hal3 orthologs. A) Recombinant GST-CaPpz1-Cter fusion protein was bound to gluthation-Sepharose 4B resin and was incubated with ScHal3 or its *C*. *albicans* orthologs CaHal3 and CaCab3 purified from *E*. *coli*. The matrix bound proteins were analyzed along with the loading controls by SDS-PAGE that was followed by Coomassie Brilliant Blue staining. The positions of the main protein bands are indicated by arrows. The calculated vs. measured molecular masses for the recombinant proteins were as follows: CaCab3 80 vs. 92 kDa, CaHal3 59 vs. 69 kDa, ScHal3 63 vs. 67 kDa and GST-CaPpz1-Cter 62 vs. 55 kDa. Note, that the proteins containing substantial intrinsically disordered domains like CaCab3 and CaHal3 exhibit slower mobility in SDS-PAGE than expected [31]. B) GST-tagged CaHal3, or CaCab3 proteins as well as GST (negative control) were immobilized to gluthation-Sepharose 4B bead and were incubated with purified 6xHis-CaPpz1. The proteins attached to the matrix and a 1:4^th^ proportions of the loaded phosphatase (loading control, first lane) were separated by SDS-PAGE and 6xHis-CaPpz1 was detected with anti-His antibody by immunoblotting. C) Either ScHal3 or its orthologues CaHal3 and CaCab3 were overexpressed with an N-terminal HA-tag in the *ppz1 S*. *cerevisiae* deletion mutant. A negative control strain was generated, that contained the empty pWS93 vector. Protein extracts were prepared from these transformed cells and were incubated with gluthation-Sepharose 4B bound GST-CaPpz1. The matrix was isolated and the attached proteins were analyzed by SDS-PAGE followed by immunoblot using anti-HA antibodies. The main immunoreavtive bands are labeled by black arrows, while the open arrow indicates a nonspecific staining.

From the structural similarities between the Hal3 family members in *S*. *cerevisiae* and *C*. *albicans* (see Introduction) we assumed that CaHal3 and CaCab3 will bind to the CaPpz1 phosphatase just as their budding yeast orthologs do [10]. To test if the proteins interacted with the conserved catalytic domain of the phosphatase we performed pull down experiments in such a way that the GST-tagged C-terminal part of the phosphatase (GST-CaPpz1-Cter) was immobilized to glutathione-Sepharose matrix and was challenged with purified recombinant proteins. Fig 2A demonstrates that CaHal3 as well as CaCab3 bind to the phosphatase containing matrix just as well as the ScHal3 that we used for comparison. However, the same approach failed when we applied it for the detection of protein complexes with the full length CaPpz1 protein, suggesting a weaker interaction between the full length phosphatase and its putative regulatory subunits. These results are reminiscent of that described for the interaction between *S*. *cerevisiae* ScPpz1 and ScHal3 [3]. In order to improve the sensitivity of our test, we resorted to detection of the tagged proteins in western blots. First we immobilized GST-CaHal3 or GST-CaCab3 to glutathione-Sepharose and incubated it with 6xHis-labeled CaPpz1. The bound proteins were subsequently detected with a 6xHis specific antibody. In a negative control experiment GST alone was bound to the matrix. It is seen in Fig 2B that the phosphatase interacted with both CaHal3 and CaCab3, and no interaction was detected in the control lane. The reciprocal setup, i.e. the immobilization of 6xHis-CaPpz1 to NTA Ni-Agarose and the detection of the GST-tagged interacting proteins with a GST specific antibody did not work out well, since for some reasons either GST-CaHal3 or GST-CaCab3 was bound to the affinity matrix even in the absence of the immobilized phosphatase, creating a strong background staining (not documented results). To circumvent the problem we retained the glutathione-Sepharose matrix but switched to HA-tagged proteins expressed in *S*. *cerevisiae* cells [15]. The interacting proteins were expressed from the pWS93 vector under the control of a strong *ADH1* promoter in a *ppz1* deletion mutant strain. Protein extracts containing HA-CaHal3 or HA-CaCab3 exhibited a strong interaction with the matrix bound GST-CaPpz1, HA-ScHal3 gave a much weaker interaction, while no signal was detected with extracts obtained from the yeast cells that were transformed with an empty pWS93 plasmid (Fig 2C). From these results collectively we conclude that in agreement with our predictions both CaHal3 and CaCab3 are able to bind to the CaPpz1 phosphatase.

### Regulation of CaPpz1 activity by CaHal3 and CaCab3

**Fig 3:**
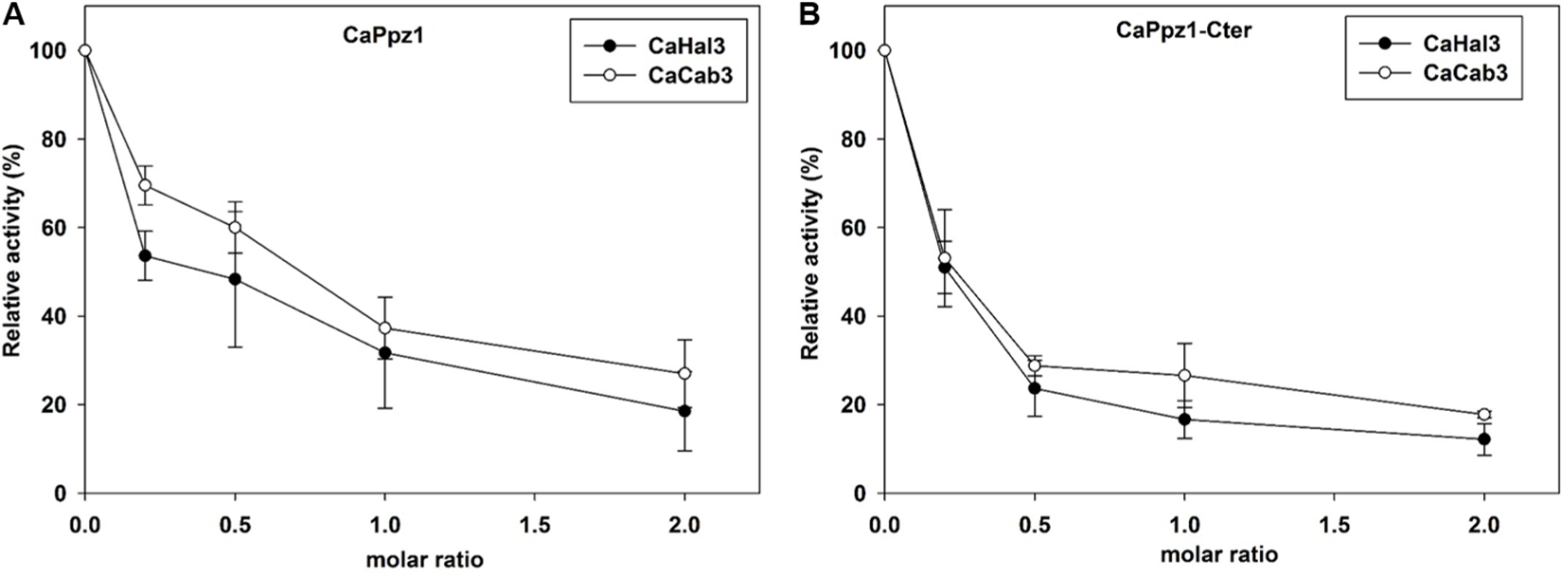
**Inhibition of CaPpz1 (A) and its catalytic domain CaPpz1-Cter (B) by CaHal3 and CaCab3 proteins.** Protein phosphatase activity was determined with [^32^P]-labeled myosin light chain substrate in the absence (100% relative activity) or in the presence of increasing concentrations of CaHal3 (●) or CaCab3 (○). The mean ± SD of three determinations are shown in this representative figure. Similar results were obtained with at least two independent sets of recombinant protein preparations.

Our next question was if the interacting proteins affected phosphatase activity. Our initial hypothesis was that only CaHal3 functions as a phosphatase regulator, but our assays proved that both CaHal3 and CaCab3 decreased the phosphatase activity in a concentration dependent manner (Fig 3). In correlation with the binding assays the catalytic domain was more sensitive to the inhibitor proteins than the full length enzyme. This was surprising, since in the case of the budding yeast proteins, ScCab3 binds to ScPpz1 but cannot inhibit the phosphatase activity [10]. Concerning the assay method we note that previously we used p-nitrophenyl phosphate substrate to determine CaPpz1 activity [18]. However this photometric assay was rather insensitive. In earlier publications a number of protein substrates of the PPZ phosphatases were tested, and trypsin digested myelin basic protein was suggested to be the most promising [32]. We found that CaPpz1 efficiently dephosphorylated ^32^P-MLC20 that was phosphorylated by myosin light chain kinase. By using this novel substrate we increased the sensitivity of the assay about 20-fold in comparison to the traditional photometric measurements. We also proved that this substrate can be used to determine the activity for ScPpz1 (S4 Fig), which is considered to be the prototype of all Ppz phosphatases. In addition, our assay is more quantitative than the one based on the mobility shift of an epitope-tagged N-terminal domain of the Reg1 protein [24]. Thus, we propose that the adaptation of the method [27] offers an alternative sensitive way for assaying the activity of this type of phosphatase with a protein substrate. Having observed an unexpected regulation of the phosphatase in the *in vitro* measurement we were curious about the functions of CaHal3 and especially CaCab3 in the living cells.

### Effects of the expression of CaHal3 and CaCab3 on Ppz-regulated functions in *S*. *cerevisiae*

**Fig 4:**
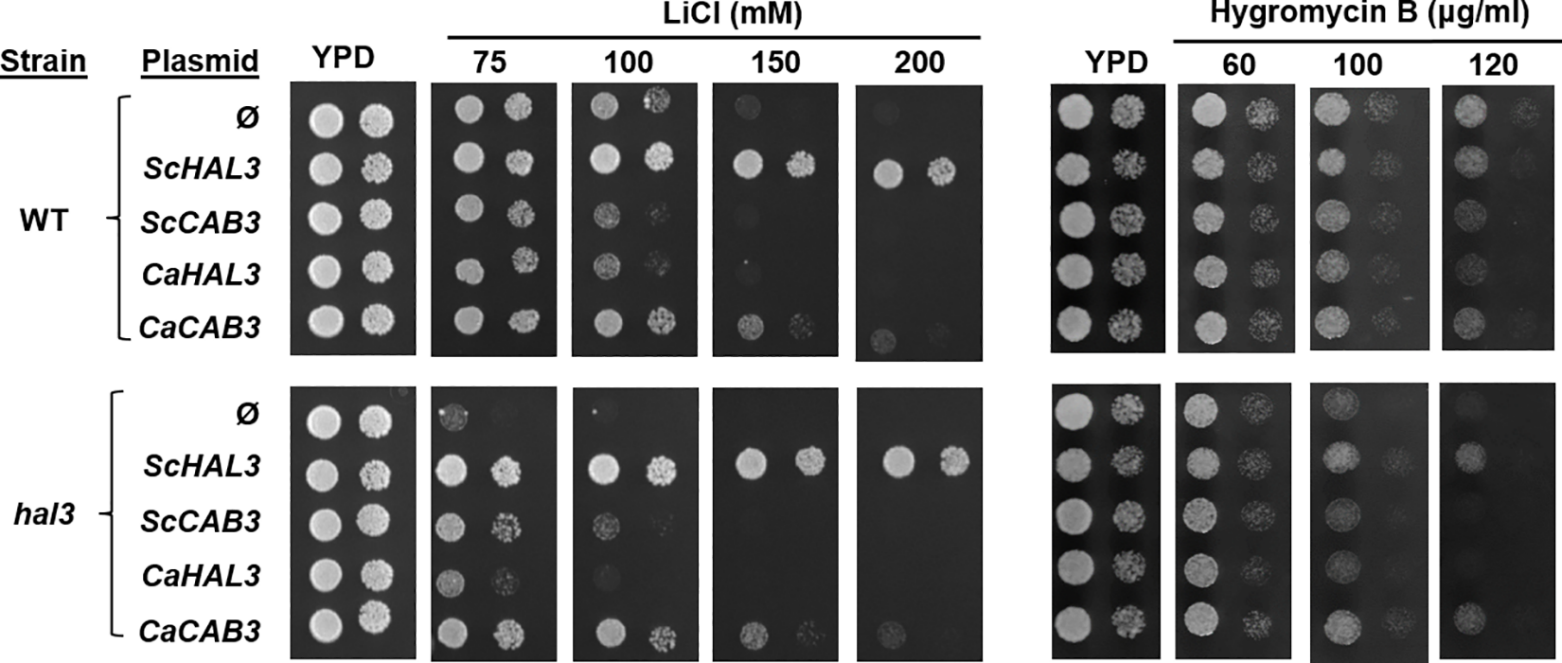
Physiological effects of CaHal3 and CaCab3 expression in wild type and *hal3 S*. *cerevisiae*. Wild type (WT) BY4741 and *hal3* mutant *S*. *cerevisiae* strains (see Table 1) were transformed with empty pWS93 plasmid (Ø) or with the same vector expressing 3xHA-tagged versions of *ScHAL3*, *ScCAB3*, *CaHAL3*, and *CaCAB3* ORFs. These transformed strains were dropped in two dilutions onto YPD plates containing no additions or increasing concentrations of LiCl and Hygromycin B. Photographs of the growing colonies were taken after 2 days of incubation.

We next investigated if CaHal3 and CaCab3 can substitute for native *S*. *cerevisiae* ScHal3 and ScCab3. To this end we first cloned both open reading frames in the episomal vector pWS93 and expressed the proteins from the constitutive *ADH1* yeast promoter. The expression of HA-tagged recombinant proteins both in wild type control and *hal3* mutant *S*. *cerevisiae* was demonstrated by western blotting (S3A Fig). High-copy expression of ScHal3 is known to increase tolerance to toxic monovalent cations, such as sodium or lithium [2] because of its inhibitory effect on ScPpz1/2 [3], whereas that of ScCab3 has no effect in these tests, or even decreases tolerance [10]. These phenotypes are explained by the negative effect of Ppz1 in the influx of potassium *via* the regulation of the high-affinity membrane transporters Trk1/Trk2 [7] and in the expression of the Na^+^-ATPase *ENA1* gene [5,6]. The consequences of the expected phosphatase inhibition were tested on the bases of these mechanisms, since it is known that the transport of potassium influences tolerance to sodium [33] and that a defect in the expression of the *ENA1* renders cells highly sensitive to toxic sodium or lithium ions [34]. As shown in Fig 4, expression of CaHal3 was unable to improve tolerance to LiCl, while unexpectedly the expression of CaCab3 increased LiCl tolerance, albeit less potently than ScHal3. This effect was even more prominent when the endogenous copy of *HAL3* was deleted (in the *hal3* mutant). It is also known that inhibition of Ppz1 decreases the electrochemical potential of the plasma membrane due to inhibition of potassium transport [29]. As a consequence, cells overexpressing ScHal3 are somewhat more resistant to toxic cations such as hygromycin B, spermine or tetramethyl ammonium [7,29]. Fig 4 shows that, in the absence of endogenous *HAL3*, overexpression of CaCab3, but not that of CaHal3, increases tolerance to hygromycin B in a similar manner to that of the expression of ScHal3. These results were surprising and indicated that (i) expression of CaHal3 was ineffective in budding yeast, and (ii) expression of CaCab3 reproduced the effects of the endogenous ScHal3 expression. The trivial technical error due to mixing or mislabeling the vectors was excluded by colony PCR (S3B Fig) indicating that nothing else just the expected products were amplified in the samples that were directly picked from the test plates. We tested whether the expected proteins were actually expressed in *S*. *cerevisiae* at the appropriate level. This was monitored by immunoblot taking advantage of the N-terminal 3xHA-tag present in the expressed proteins. As shown in S3A Fig both CaHal3 and CaCab3 were expressed in different yeast strains at levels similar to that observed for the budding yeast counterparts. Therefore, the failure of CaHal3 substituting for ScHal3 cannot be attributed to deficient expression of the former. Finally, we investigated if the *C*. *albicans* proteins affected the *S*. *cerevisiae* phosphatase activity *in vitro*. We found that both CaHal3 and CaCab3 inhibited either the catalytic domain (ScPpz1-Cter) or the full length (ScPpz1) forms of the heterologous phosphatase (S4 Fig), although less effectively than the homologous *Candida* enzyme forms (Fig 3). Thus, we have to accept the fact that both CaHal3 and CaCab3 can inhibit the PPZ phosphatases but only one of them, namely CaCab3 can partially reproduce the physiological effect of ScHal3 *in vivo*. To underpin these results, that contradicted simple structural predictions, we studied the effects of the *C*. *albicans* proteins on additional Ppz related functions in *S*. *cerevisiae*.

**Fig 5:**
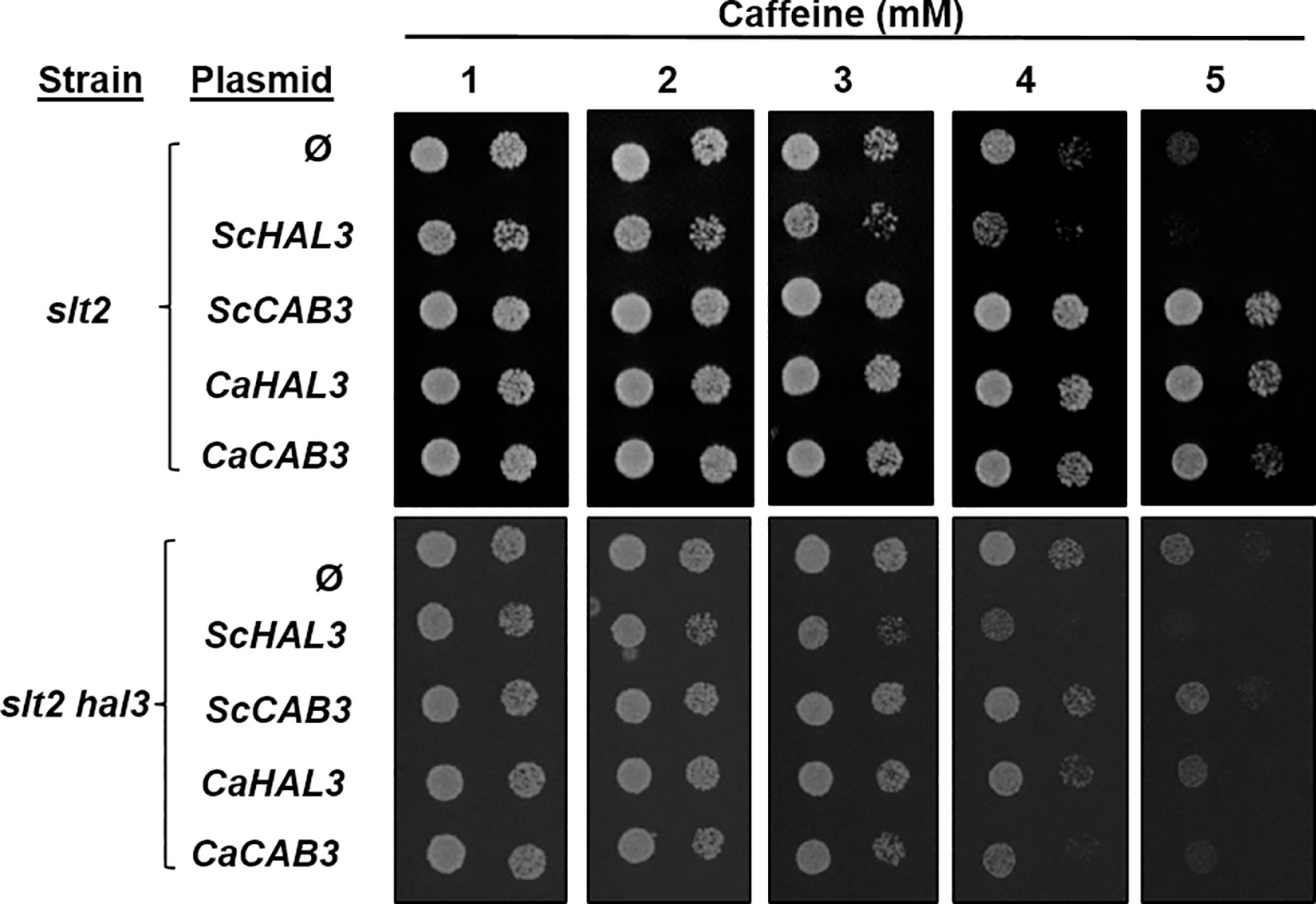
Physiological effects of CaHal3 and CaCab3 expression in *slt2* and *slt2 hal3 S*. *cerevisiae* mutants. The vectors described in Fig 4 were transformed into *slt2* or *slt2 hal3 S*. *cerevisiae* mutant strains. Transformants were cultivated on synthetic media (ura-) plates containing either no additions or increasing concentrations of caffeine. Photographs were taken after 3 days of incubation.

**Fig 6:**
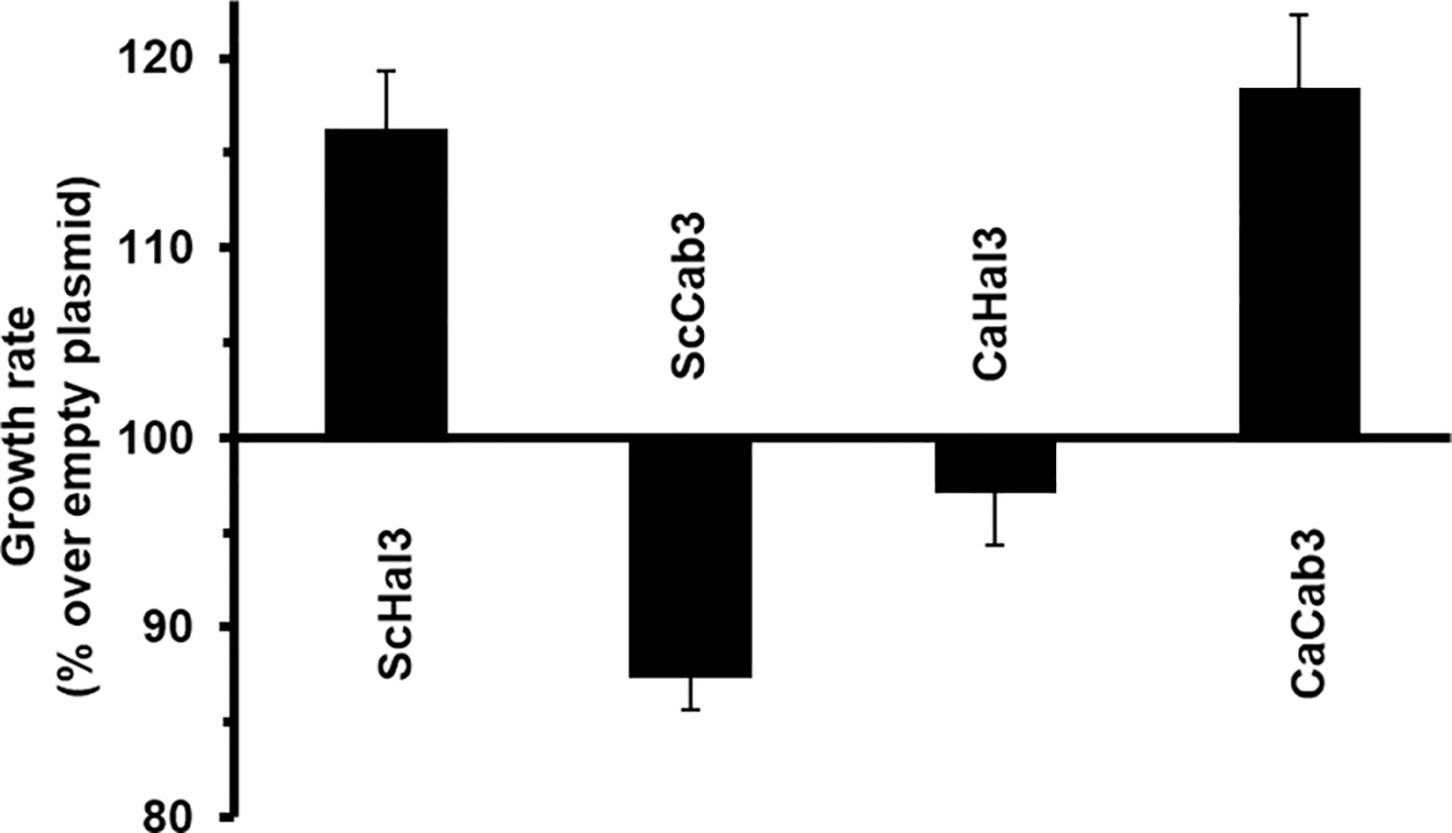
Effect of expression of CaHal3 and CaCab3 on growth at limiting potassium conditions. The strain *hal3*:*kanMX4* was transformed with the indicated plasmids (see Fig 4) and inoculated at OD_600_ = 0.004–0.008 in Translucent K^+^-free medium [28] supplemented with 1 mM (low) or 50 mM (high) KCl. Growth was resumed for 16 h and the low/high K^+^ OD_600_ ratio was calculated. The values were referred to the ratio observed for the strain carrying the empty plasmid, which is taken as 100. Data are the means ± SEM from 9 experiments performed by triplicate.

The increase of turgor pressure caused by inactivation of Ppz phosphatases activates the Slt2 MAP kinase pathway, indicating that it represents a form of cell-wall stress [35]. Consequently, cells mutated for *PPZ* genes [36] or overexpressing ScHal3 [14] are sensitive to caffeine. This sensitivity is exacerbated in the absence of the Slt2 kinase, which is necessary to adapt to cell-wall stress conditions. We show in Fig 5 that expression of CaCab3, but not that of CaHal3, decreases tolerance to caffeine in the *slt2 hal3* strain, pointing again to the notion that CaCab3 is performing Hal3-like functions in *S*. *cerevisiae*. The mentioned increase in turgor pressure is likely the result of increased influx of potassium, an effect derived from overexpression of ScHal3 *via* inhibition of Ppz phosphatases [2,7]. Alterations in potassium availability can be readily detected by changes in growth rate when the concentration of the cation in the medium becomes limiting. Therefore, we decided to determine the growth rate of *hal3* cells expressing Hal3 and Cab3 from both *S*. *cerevisiae* and *C*. *albicans* using the ratio of growth at limiting (1 mM) or plenty (50 mM) potassium as reference. As shown in Fig 6, overexpression of ScHal3, as expected, increased the growth ratio in comparison with cells harboring an empty plasmid, whereas expression of ScCab3 resulted in poorer growth at limiting potassium. This is not surprising since, as mentioned above, ScCab3 binds to Ppz1 but cannot inhibit the phosphatase. Therefore, an excess of ScCab3 likely interferes with endogenous Vhs3 in these cells, resulting in higher-than-normal Ppz1 activity and increased potassium influx. Remarkably, overexpression of CaCab3 increased the growth ratio similarly to that observed for ScHal3, whereas expression of CaHal3 had a slightly negative effect. Therefore, these results fully confirm the notion that, in *S*. *cerevisiae*, CaCab3 can carry out the functions related to regulation of Ppz1 that endogenous ScHal3 does, but CaHal3 cannot. This conclusion is in agreement with the moderately higher *in vitro* inhibitory capacity of CaHal3 towards both the full length phosphatase and its catalytic C-terminal domain (S4 Fig).

### The role of CaHal3 and CaCab3 in PPCDC function

As described in the Introduction, PPCDC activity in *S*. *cerevisiae* requires at least two gene products, Hal3 (or Vhs3) and Cab3 to build a functional heterotrimeric enzyme. To test the relative contribution of CaHal3 and CaCab3 to this function, we expressed each *C*. *albicans* and *S*. *cerevisiae* protein in diploid heterozygous *S*. *cerevisiae* strains lacking *CAB3* or *HAL3* and *VHS3*, induced the sporulation of these strains, and tested by tetrad analysis the ability for growing of the haploid derivatives. As shown in Table 2, and as previously demonstrated [10], ScHal3 eliminated the synthetic lethality of the *hal3* and *vhs3* mutations, whereas ScCab3 did not. In contrast, expression of ScCab3 complemented the *cab3* mutant, but not the *hal3 vhs3* one. On the other hand, expression of CaHal3 allowed growth of the haploid *hal3 vhs3* strain, but not that of the *cab3* mutant, whereas the opposite behavior was observed for the expression of the CaCab3. Therefore, and in contrast to that observed in the previous section, each *Candida* protein performs as their *Saccharomyces* counterpart, as far as the PPCDC function is concerned. This is interesting because it implies that, even though CaCab3 contains a His residue equivalent to the catalytic His^378^ in ScHal3, it must not be functional, like in ScCab3. A possible explanation would be that CaCab3 contains a sequence insert preceding the relevant His (His^466^) even larger than that observed in ScCab3 (S5 Fig). Such insert was found to be crucial for the inability of this His to serve as catalytic residue [10]. The failure of CaHal3 to complement the *cab3* mutation in *S*. *cerevisiae* was not surprising, due to the absence of the catalytic Cys residue in the position equivalent to Cys^478^ in ScCab3 (Fig 1).

**Table 2:** Complementation of PPCDC function in *S*. *cerevisiae* by *C*. *albicans* CaHal3 and CaCab3.

Plasmids	pWS93	ScHal3	ScCab3	CaHal3	CaCab3
***Strains***					
***cab3***	No (9)	No (9)	Yes (9)	No (9)	Yes (9)
***hal3 vhs3***	No (19)	Yes (17)	No (12)	Yes (35)	No (24)

See text for details. Numbers between parentheses denote the number of tetrads analyzed in each case.

## Conclusions

Our present study was initiated on the basis of structural predictions suggesting a functional similarity between the orthologous *C*. *albicans* proteins, CaHal3 and CaCab3, and their *S*. *cerevisiae* counterparts ScHal3/ScVhs3 and ScCab3 (Fig 1). However, our expectations were only partially fulfilled. The strict conservation of PPCDC related functions of these proteins is evident from our tetrad analysis: CaHal3 compensates the absence of the ScHal3 and ScVhs3 proteins together, while CaCab3 can replace ScCab3 in the enzyme reaction that is essential for CoA biosynthesis (Table 2). From this result it can be deduced (i) that the conserved His466 in CaCab3 is not functional (like His391 in ScCab3) since this protein cannot rescue the lethality of the *hal3 vhs3* double mutant, and (ii) that orf19.7378 (*CaHAL3*) and orf19.3260 (*CaCAB3*) are essential genes. These conclusions are in complete agreement with the experimental evidence and the conservation of the amino acid sequences that are related to the PPCDC activity (S5 Fig).

On the contrary, the PPZ phosphatase related functions of the same proteins turned out to be unpredictable from the primary structures: only the expression of CaCab3 was able to replace to a considerable extent the tested phosphatase related functions of ScHal3 in *S*. *cerevisiae* mutants, while the structurally more similar CaHal3 was ineffective (Figs 4–6), even if *in vitro* assays indicate that both proteins are equally potent inhibitors of the Ppz phosphatases (Fig 3 and S4 Fig). The molecular bases for such an unexpected behavior are presently unclear. By mutagenesis analysis, Munoz and coworkers [14] identified 9 amino acid residues that were required for the inhibition of the ScPpz1 phosphatases by ScHal3. In S5 Fig it is seen, that 7 out of these residues were conserved in the *Candida* ortholog CaHal3 while only 2 of them were identical in CaCab3. Therefore, conservation of the selected residues cannot be taken as a discriminatory factor. It is possible that the phosphatase inhibition is not exclusively determined by the amino acids that were found inside or close to the PPCDC active site interfaces [14], but other residues which fall into the intrinsically disordered acidic C-terminal segments or in the disordered N-terminal region are also involved. These regions have been shown to contribute to the regulatory function of Hal3 on Ppz1 in *S*. *cerevisiae* [15]. However, this assumption is weakened by the observation that most of the fungal Hal3/Cab3-like proteins (excluding the *Saccharomycetales* and, interestingly, the *Ustilaginales*) lack the N-terminal and acidic C-terminal extensions that flank the PD core in the *Candida* and *Saccharomyces* orthologs as shown in Fig 1. Certainly, the understanding of the evolution and functioning of these additional intrinsically disordered segments in the PPCDC subunits of the *Saccharomycetales* and *Ustilaginale*s deserves more investigations. The lack of the physiological effects of CaHal3 in *S*. *cerevisiae* cells is even more difficult to reconcile on a solid structural basis. It is possible that CaHal3 incorporates into the *S*. *cerevisiae* PPCDC complex more effectively than its paralog, but we cannot exclude the possibility that the unique N-terminal segment of this protein directs it towards a presently unknown partner, that prevents its effective interaction with the ScPpz1/2 phosphatases. Alternatively, as shown in S5 Fig, ScHal3 and CaCab3 contain an insert of significant size (30 residues), before the conserved His, that is not present in ScCab3 or CaHal3. It can be hypothesized that such inserts generate some kind of secondary structure that is important to stabilize *in vivo* interactions with Ppz1. Hopefully, our undergoing structure-function studies will reveal the exact mechanism of the Hal3-Ppz1/2 interactions.

In conclusion, in *C*. *albicans* we identified two essential subunits of the PPCDC enzyme (CaHal3 and CaCab3) and demonstrated that at least one of them (CaCab3) could be a physiologically relevant negative regulator of the fungus specific protein phosphatase Z. While our structure-driven expectations were proven, as far as the essential functions of the oligomeric decarboxylase enzyme are concerned, we made a number of unpredictable observations when the non-essential, phosphatase-related moonlighting function of the two proteins was investigated. Our results shed light on a possible mechanism of phosphatase regulation and will guide further studies in the direction of exploring the exact physiological roles of the PPCDC subunits in *C*. *albicans*.

## Supporting Information

S1 Fig The phylogenetic relationships between fungal PPCDC subunits. The evolutionary history of the proteins was inferred using the Neighbor-Joining method [S1]. The optimal tree with the sum of branch length = 5.12386958 is shown. The percentage of replicate trees in which the associated taxa clustered together in the bootstrap test (500 replicates) are shown next to the branches [S2]. The tree is drawn to scale, with branch lengths in the same units as those of the evolutionary distances used to infer the phylogenetic tree. The evolutionary distances were computed using the Poisson correction method [S3] and are in the units of the number of amino acid substitutions per site. The analysis involved 12 PPCDC amino acid sequences of *Saccharomyces cerevisiae* (ScHal3, ScVhs3, ScCab3), *Candida albicans* (CaHal3, CaCab3), *Schizosaccharomyces pombe* (SpHal3), *Kluyveromyces lactis* (KlHal3, KlCab3), *Yarrowia lipolytica* (YlHal3, YlCab3), and *Zygosaccharomyces rouxi* (ZrHal3, ZrCab3), as well as 2 thymidylate synthase sequences (ScCdc21 and CaCdc21) for comparison. All positions containing gaps and missing data were eliminated. There were a total of 208 positions in the final dataset. Evolutionary analyses were conducted in MEGA6 [S4]. The three distinct branches of the three, namely the Hal3-like proteins, the Cab3-like proteins and the unorthodox thymidylate synthase like proteins are highlighted in different colors.
(TIF)

S2 Fig SDS-PAGE characterization of the recombinant protein preparations that were used in the present study. The purified proteins either in solution or immobilized to affinity matrices, as required, were separated in a 12% SDS-polyacrylamide gel and stained with Coomassie Blue. The main bands corresponding to the recombinant proteins are boxed. The calculated vs. measured molecular mass values for each recombinant protein are given in parentheses. The molecular mass standards (St) are labeled by arrows indicating their sizes in kDa. Note, that the proteins containing large intrinsically disordered segments like CaHal3 and CaCab3 exhibit anomalous mobility in SDS-PAGE [S5].
(TIF)

S3 Fig Western blot and colony PCR analyses of *S*. *cerevisiae* strains expressing Hal3/Cab3 proteins. (A) Wild type BY4741 and *hal3* deletion mutant *S*. *cerevisiae* strains were transformed with pWS93 based plasmids, as described in Fig 4. The overexpressed HA-tagged recombinant proteins ScHal3, ScCab3, CaHal3, and CaCab3 were detected with HA specific antibody by western blotting. The main immunoreactive bands are labeled by arrows. (B) The presence of the CaHal3 or CaCab3 sequence carrying plasmids was verified with colony PCR after drop test. Clones were directly harvested from YPD plates containing the *hal3* mutant cells and were analyzed by using either the CaCAB3 specific CaCab3EcoRI-CaCab3XhoI primers or the CaHAL3 specific CaHal3-EcoRI-CaHal3XhoI primers (S1 Table) in the PCR. The samples are identified as in Fig 4. The sizes of the standards (St) and the amplicons are shown in base pairs (bp).
(TIF)

S4 Fig **Inhibition of ScPpz1 (A) and its catalytic domain ScPpz1-Cter (B) by CaHal3 and CaCab3 proteins.** The effect of CaHal3 (●) or CaCab3 (◯) on the activity of recombinant *S*. *cerevisiae* phosphatases was investigated as described in Fig 3. The mean ± SD of three assays is shown.
(TIF)

S5 Fig Sequence comparison of the PD domains of *S*. *cerevisiae* and *C*. *albicans* Hal3 and Cab3 proteins. *A*. *thaliana* Hal3a is included for reference. The PD domains are defined in Fig 1. Underlined residues indicate the essential His in Hal3, the essential Cys in Cab3, and the Asn motif found in Cab3 proteins. Mutations in ScHal3 reported in [S6] are shown in a yellow background. Italicized sequences correspond to the inserts found in ScHal3 and CaCab3 just before the conserved His.
(TIF)

S1 References Supplementary references. (DOCX)

S1 Table Oligonucleotide primers used for cloning. (DOCX)
