# Supplementary figures and images for: Analysis of Two Putative Candida albicans Phosphopantothenoylcysteine Decarboxylase / Protein Phosphatase Z Regulatory Subunits Reveals an Unexpected Distribution of Functional Roles
Source: PLoS One. 2016 Aug 9;11(8):e0160965. doi: 10.1371/journal.pone.0160965 (PMC4978486; doi:10.1371/journal.pone.0160965)

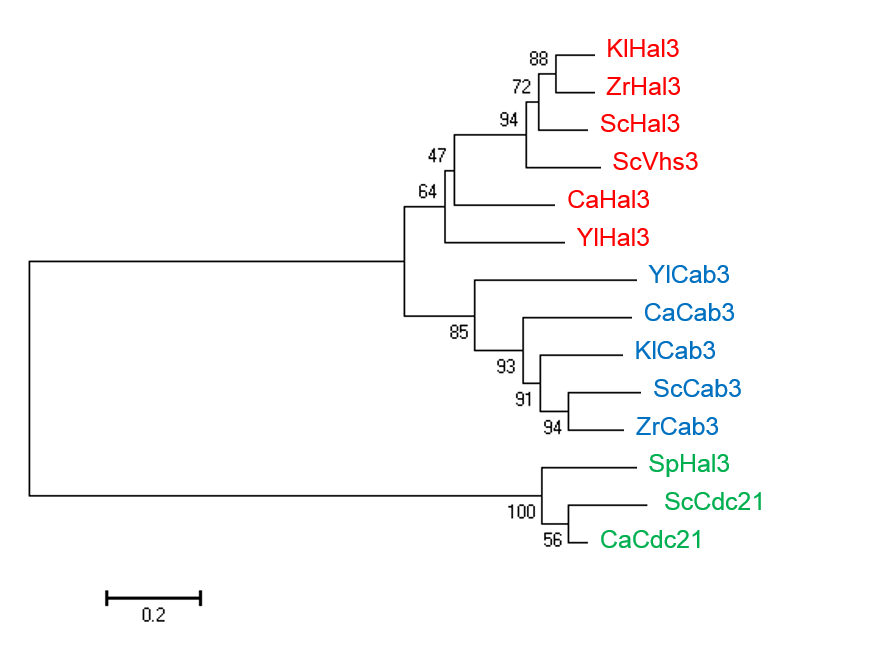

Supplement: S1 Fig — The evolutionary history of the proteins was inferred using the Neighbor-Joining method [S1]. The optimal tree with the sum of branch length = 5.12386958 is shown. The percentage of replicate trees in which the associated taxa clustered together in the bootstrap test (500 replicates) are shown next to the branches [S2]. The tree is drawn to scale, with branch lengths in the same units as those of the evolutionary distances used to infer the phylogenetic tree. The evolutionary distances were computed using the Poisson correction method [S3] and are in the units of the number of amino acid substitutions per site. The analysis involved 12 PPCDC amino acid sequences of Saccharomyces cerevisiae (ScHal3, ScVhs3, ScCab3), Candida albicans (CaHal3, CaCab3), Schizosaccharomyces pombe (SpHal3), Kluyveromyces lactis (KlHal3, KlCab3), Yarrowia lipolytica (YlHal3, YlCab3), and Zygosaccharomyces rouxi (ZrHal3, ZrCab3), as well as 2 thymidylate synthase sequences (ScCdc21 and CaCdc21) for comparison. All positions containing gaps and missing data were eliminated. There were a total of 208 positions in the final dataset. Evolutionary analyses were conducted in MEGA6 [S4]. The three distinct branches of the three, namely the Hal3-like proteins, the Cab3-like proteins and the unorthodox thymidylate synthase like proteins are highlighted in different colors. (TIF) [file pone.0160965.s001.tif]

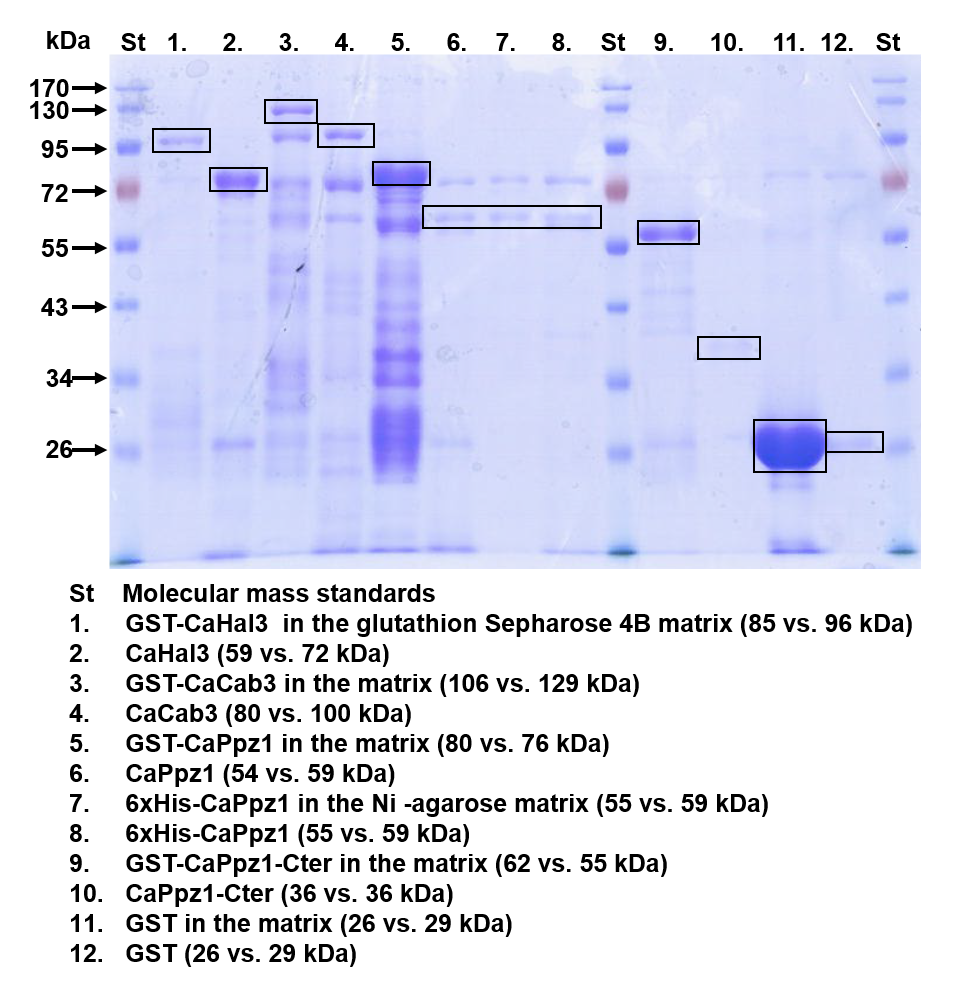

Supplement: S2 Fig — The purified proteins either in solution or immobilized to affinity matrices, as required, were separated in a 12% SDS-polyacrylamide gel and stained with Coomassie Blue. The main bands corresponding to the recombinant proteins are boxed. The calculated vs. measured molecular mass values for each recombinant protein are given in parentheses. The molecular mass standards (St) are labeled by arrows indicating their sizes in kDa. Note, that the proteins containing large intrinsically disordered segments like CaHal3 and CaCab3 exhibit anomalous mobility in SDS-PAGE [S5]. (TIF) [file pone.0160965.s002.tif]

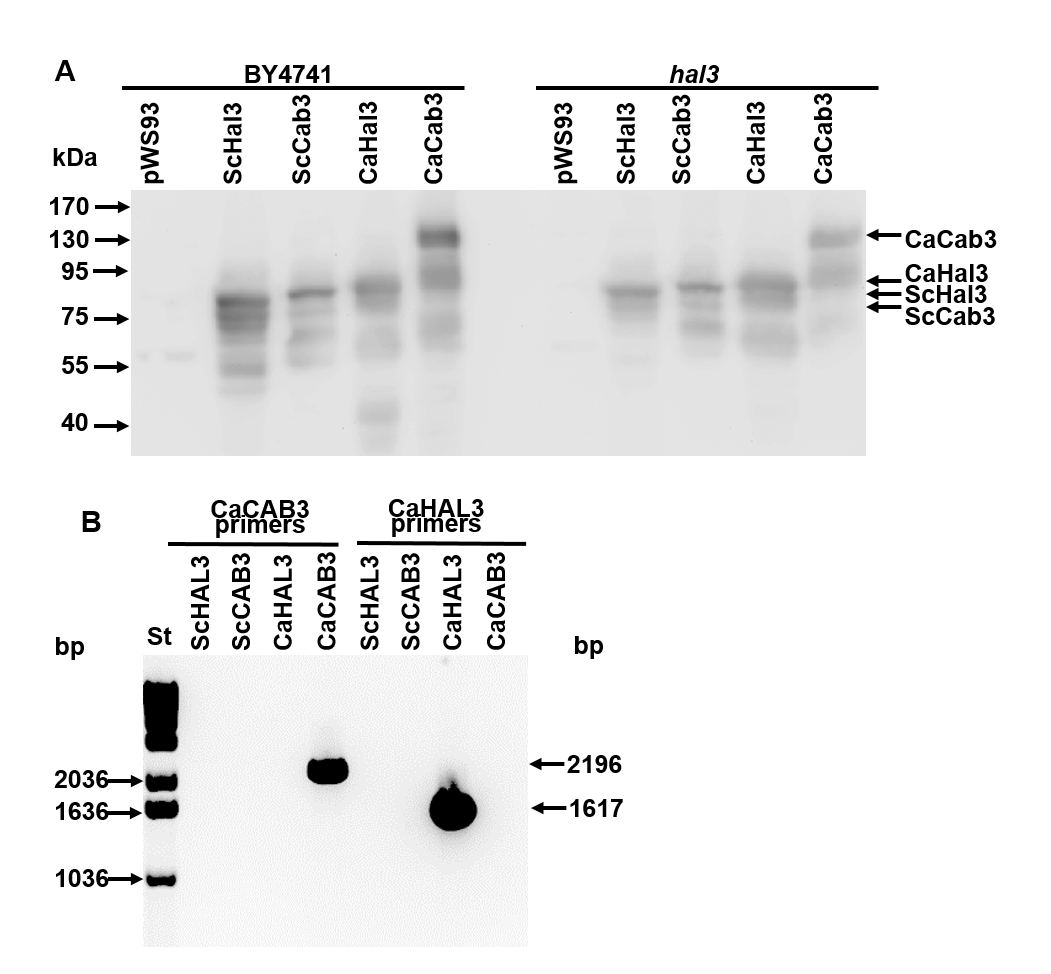

Supplement: S3 Fig — (A) Wild type BY4741 and hal3 deletion mutant S. cerevisiae strains were transformed with pWS93 based plasmids, as described in Fig 4. The overexpressed HA-tagged recombinant proteins ScHal3, ScCab3, CaHal3, and CaCab3 were detected with HA specific antibody by western blotting. The main immunoreactive bands are labeled by arrows. (B) The presence of the CaHal3 or CaCab3 sequence carrying plasmids was verified with colony PCR after drop test. Clones were directly harvested from YPD plates containing the hal3 mutant cells and were analyzed by using either the CaCAB3 specific CaCab3EcoRI-CaCab3XhoI primers or the CaHAL3 specific CaHal3-EcoRI-CaHal3XhoI primers (S1 Table) in the PCR. The samples are identified as in Fig 4. The sizes of the standards (St) and the amplicons are shown in base pairs (bp). (TIF) [file pone.0160965.s003.tif]

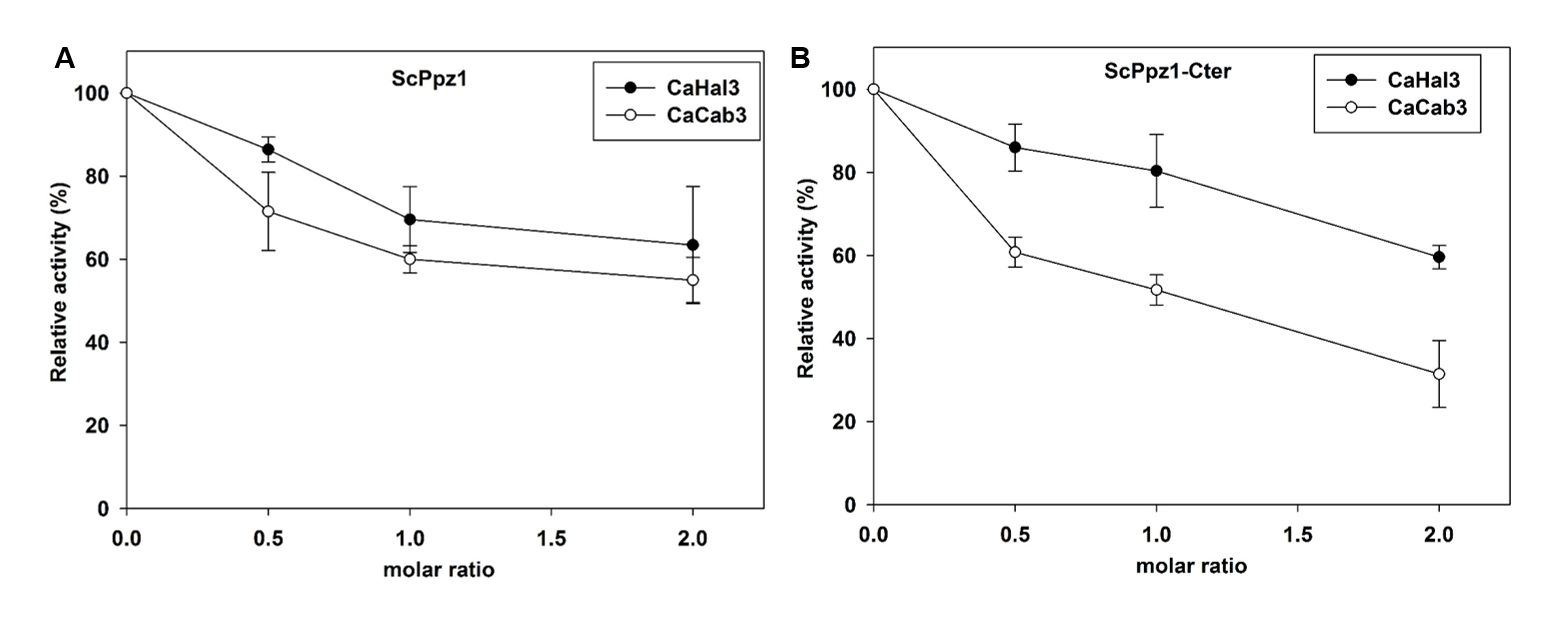

Supplement: S4 Fig — Inhibition of ScPpz1 (A) and its catalytic domain ScPpz1-Cter (B) by CaHal3 and CaCab3 proteins. The effect of CaHal3 (●) or CaCab3 (◯) on the activity of recombinant S. cerevisiae phosphatases was investigated as described in Fig 3. The mean ± SD of three assays is shown. (TIF) [file pone.0160965.s004.tif]

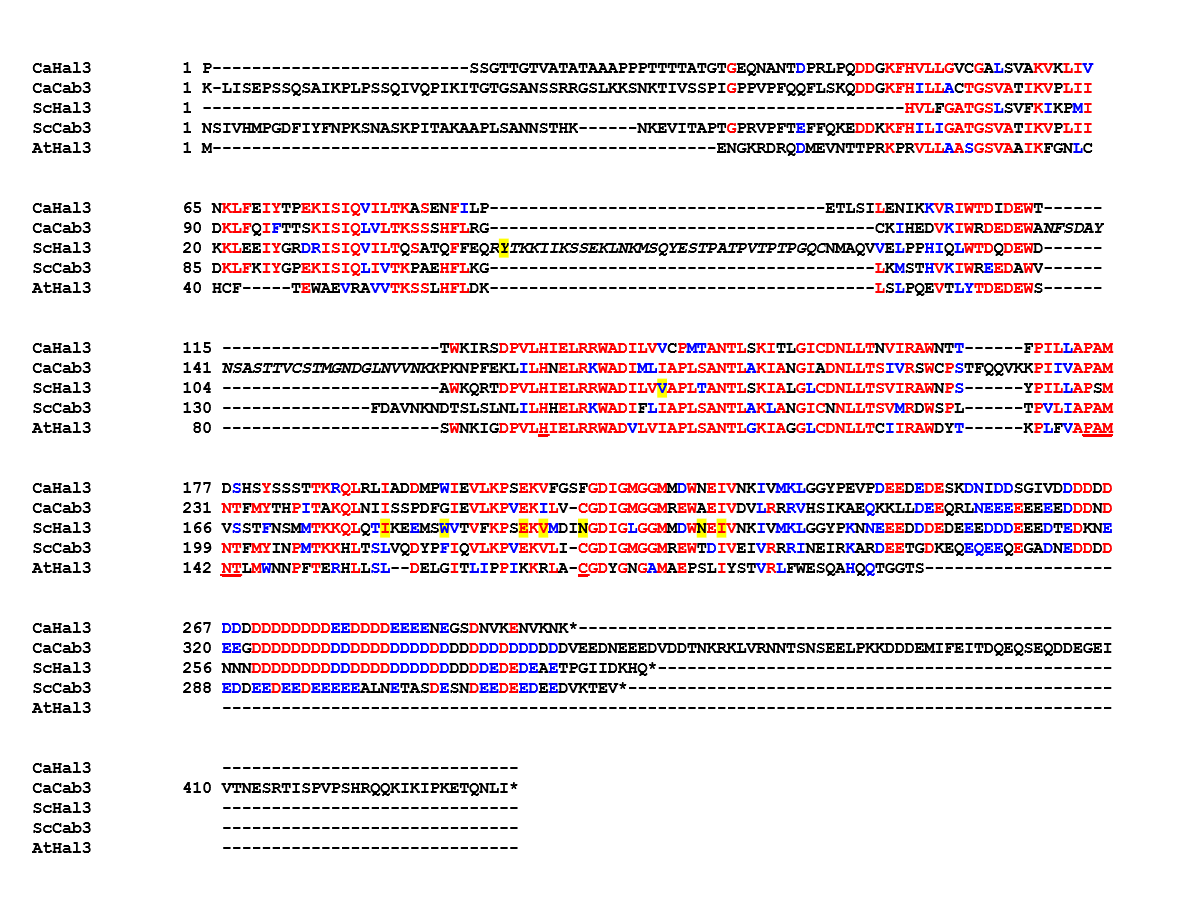

Supplement: S5 Fig — A. thaliana Hal3a is included for reference. The PD domains are defined in Fig 1. Underlined residues indicate the essential His in Hal3, the essential Cys in Cab3, and the Asn motif found in Cab3 proteins. Mutations in ScHal3 reported in [S6] are shown in a yellow background. Italicized sequences correspond to the inserts found in ScHal3 and CaCab3 just before the conserved His. (TIF) [file pone.0160965.s005.tif]
