# Supplementary material for: Analysis of Two Putative Candida albicans Phosphopantothenoylcysteine Decarboxylase / Protein Phosphatase Z Regulatory Subunits Reveals an Unexpected Distribution of Functional Roles
Source: PLoS One. 2016 Aug 9;11(8):e0160965. doi: 10.1371/journal.pone.0160965 (PMC4978486; doi:10.1371/journal.pone.0160965)
Supplement: S1 References — (DOCX) [file pone.0160965.s006.docx]

**Supplementary references**

S1. Saitou N. and Nei M. The neighbor-joining method: A new method for reconstructing phylogenetic trees. *Molecular Biology and Evolution* 1987; 4:406-425

S2. Felsenstein J. Confidence limits on phylogenies: An approach using the bootstrap. *Evolution* 1985;39:783-791

S3. Zuckerkandl E. and Pauling L. Evolutionary divergence and convergence in proteins. Edited in *Evolving Genes and Proteins* by V. Bryson and H.J. Vogel, 1965;pp. 97-166. Academic Press, New York.

S4. Tamura K., Stecher G., Peterson D., Filipski A., and Kumar S. MEGA6: Molecular Evolutionary Genetics Analysis version 6.0. *Molecular Biology and Evolution* 2013;30: 2725-2729

S5. Tompa P. Intrinsically unstructured proteins. *Trends Biochem. Sci.* 2002; 27:527-533

S6. Munoz I, Ruiz A, Marquina M, Barcelo A, Albert A, Arino J. Functional characterization of the yeast Ppz1 phosphatase inhibitory subunit Hal3: a mutagenesis study*. J Biol Chem* 2004;279:42619-27
