# Supplementary material for: Analysis of Two Putative Candida albicans Phosphopantothenoylcysteine Decarboxylase / Protein Phosphatase Z Regulatory Subunits Reveals an Unexpected Distribution of Functional Roles
Source: PLoS One. 2016 Aug 9;11(8):e0160965. doi: 10.1371/journal.pone.0160965 (PMC4978486; doi:10.1371/journal.pone.0160965)
Supplement: S1 Table — (DOCX) [file pone.0160965.s007.docx]

**S1 Table. Oligonucleotide primers used for cloning**

| Name | Sequence, 5’-3’ | Target |
| --- | --- | --- |
| Hal3-200 | GAAGTCACAGAGTGCTCTTGG | *hal3::KanMX4*  genomic DNA |
| Hal3-3’_term1 | GTGACATCGATACCTTCACTTAC | *hal3::KanMX4*  genomic DNA |
| CaHAL3*Eco*RI | AATT*GAATTC***ATG**CCTTCTGATACTCCCAAAG | CaHAL3 ORF |
| CaHAL3*Xho*I | TCAT*CTCGAG*TCATTTATTCTTGACATTCTCTTTAAC | CaHAL3 ORF |
| CaCab*Eco*RI | AATA*GAATTC***ATG**TCCCAAGATTCTTCAGATC | CaCAB3 ORF |
| CaCab*Xho*I | TTAT*CTCGAG*TCAAATAAGATTTTGAGTTTCTTTAGG | CaCAB3 ORF |
| CaPpz1*Eco*RI | ATA*GAATTC***ATG**GGTTCTAATTCA | CaPPZ1-3 ORF |
| CaPpz1*Xho*I | CCT*CTCGAG*CTTTATGTAGATTC | CaPPZ1-3 ORF |
| CaPPZ-Cter*Bam*HI | AAA*GGATCC*ATAGATATAGATTCATTAATTGATAAATTA | CaPPZ1-3 ORF |
| CaPPZ-Cter*Xho*I | TTTA*CTCGAG*CTTTATGTAGATTTCTTTC | CaPPZ1-3 ORF |

The engineered restriction sites are written in italics, initiating ATG codons are shown in bold, while STOP codons are underlined.
